# LRPPRC facilitates tumor progression and immune evasion through upregulation of m^6^A modification of PD-L1 mRNA in hepatocellular carcinoma

**DOI:** 10.3389/fimmu.2023.1144774

**Published:** 2023-03-30

**Authors:** Houhong Wang, Amao Tang, Yayun Cui, Huihui Gong, Heng Li

**Affiliations:** ^1^ Department of General Surgery, The Affiliated Bozhou Hospital of Anhui Medical University, Bozhou, Anhui, China; ^2^ Department of Gastroenterology, The Affiliated Hangzhou First People’s Hospital, Zhejiang University School of Medicine, Hangzhou, Zhejiang, China; ^3^ Department of Cancer Radiotherapy, The First Affiliated Hospital of USTC, Division of Life Sciences and Medicine, University of Science and Technology of China (Anhui Provincial Cancer Hospital), Hefei, Anhui, China; ^4^ Faculty of Health and Life Sciences, Oxford Brookes University, Oxford, United Kingdom; ^5^ Department of Comprehensive Surgery, Anhui Provincial Cancer Hospital, West District of The First Affiliated Hospital of USTC, Hefei, Anhui, China

**Keywords:** hepatocellular carcinoma, LRPPRC, PD-L1, immune evasion, m6A modification, anti-tumor immunity, tumor progression

## Abstract

**Objective:**

LRPPRC is a newly discovered N^6^-methyladenosine (m^6^A) modification reader, which potentially affects hepatocellular carcinoma (HCC) progression. PD-L1 in tumor cells is essential for tumor immune evasion. This work investigated the LRPPRC-mediated m^6^A-modification effect on PD-L1 mRNA and immune escape in HCC.

**Methods:**

Expression and clinical implication of LRPPRC and PD-L1 were measured in human HCC cohorts. The influence of LRPPRC on malignant behaviors of HCC cells was investigated through *in vitro* assays and xenograft tumor murine models. The posttranscriptional mechanism of LRPPRC on PD-L1 and anti-tumor immunity was elucidated in HCC cells *via* RIP, MeRIP−qPCR, RNA stability, immunohistochemical staining, and so forth.

**Results:**

LRPPRC exhibited the notable upregulated in human HCC tissues, which was in relation to advanced stage and worse overall survival and disease-free survival. Impaired proliferative capacity and G2/M phage arrest were found in LRPPRC-knockout cells, with increased apoptotic level, and attenuated migratory and invasive abilities. In HCC patients and murine models, LRPPRC presented a positive interaction with PD-L1, with negative associations with CD8+, and CD4+ T-cell infiltrations and chemokines CXCL9, and CXCL10. LRPPRC loss downregulated the expression of PD-L1 and its m^6^A level in HCC cells. Moreover, LRPPRC suppression mitigated tumor growth in murine models and improved anti-tumor immunity and immune infiltration in tumors.

**Conclusion:**

This work unveiled that LRPPRC may posttranscriptionally upregulate PD-L1 partially with an m^6^A-dependent manner for heightening mRNA stabilization of PD-L1 and provided a new mechanism for m^6^A regulator-mediated immunosuppression in HCC.

## Introduction

Globally, hepatocellular carcinoma (HCC) remains one of the most prevalent causes of cancer-associated deaths (~800,000 cases per year) (1). Less than 20% of HCC patients survive over 1 year following initial diagnosis (2). Liver transplantation brings the optimal first-rank outcomes for patients who meet strict criteria (3). Immunotherapy with checkpoint inhibitors has revolutionized the clinical management of unresectable HCC. Single-agent anti-programmed death-1 (PD-1) inhibitor has exhibited a promising efficacy against HCC in early phase clinical trials (4). Atezolizumab [anti-programmed death ligand 1 (PD-L1) antibody] in combination with bevacizumab (anti-VEGF antibody) has gained the approval as the first-line setting, which can improve overall survival (5). Moreover, durvalumab (anti–PD-L1 agent) combined with tremelimumab (anti-CLTA4 agent) has displayed the superiority in prolonging overall survival time (6). Single-agent pembrolizumab (anti–PD-1 antibody) (7) and the combination of nivolumab (anti–PD-1 antibody) with ipilimumab (anti-CLTA4 antibody) (8) have been approved as the second-line therapeutic options. While immunotherapy has achieved such major advances, the molecular basis for controlling immune response and escape remains indistinct.

N^6^-methyladenosine (m^6^A) remains the most abundant RNA modification type in humans, which affects almost every process of mRNA metabolism (9). This modification is installed *via* methyltransferase complex and removed *via* demethylases (10, 11). In addition, the m^6^A reader proteins are capable of recognizing the m^6^A-modified RNAs (12). Leucine rich pentatricopeptide repeat containing (LRPPRC) is a newly discovered reader of m^6^A modification, which is frequently overexpressed in HCC tissue (13), and its overexpression is related to unfavorable prognostic outcomes of HCC (14). As for molecular mechanisms, LRPPRC can sustain Yap-P27–induced cell ploidy and P62-HDAC6–controlled autophagy maturation as well as attenuate genome instability and HCC progression (15). In addition, through interacting with LRPPRC, lncRNA SNHG17 stabilizes c-Myc protein and facilitates G1/S transition and cellular proliferation in HCC (16). Limited evidence proves that LRPPRC enables to mediate immunity or immune response. LRPPRC presents a negative correlation to most tumor-infiltrating immune cells in lung adenocarcinoma (17). In periodontitis, MHC molecules HLA-B and HLA-DOA can be potentially affected by LRPPRC (18). In HCC, LRPPRC upregulation correlates to decreased T cells, cytotoxic cells, dendritic cells as well as cytolytic activity response (19). In addition, it exhibits a positive relationship with PD-L1 (20). On the basis of existing evidence, this work offers a novel posttranscriptional mechanism by which LRPPRC facilitates tumor progression as well as mediates immune evasion in HCC through elevating m^6^A modification of PD-L1 mRNA, which might offer a possible strategy for the immunotherapy against HCC *via* utilizing LRPPRC as a therapeutic target.

## Materials and methods

### Bioinformatics analysis

Through utilizing the TIMER2.0 platform (http://timer.cistrome.org/) (21), pan-cancer analysis was conducted on LRPPRC expression in tumors and control tissue specimens. Its expression was also measured in HCC and control tissues *via* the GEPIA2 web server (http://gepia2.cancer-pku.cn/#index) (22). The difference in LRPPRC expression among distinct tumor stages was assessed in HCC patients. Overall survival and disease-free survival probabilities of patients with lowly or highly expressed LRPPRC were plotted as Kaplan–Meier curves, followed by log-rank test.

### Tissue specimens

In total, 30 paired HCC and adjacent normal tissues were gathered from the Anhui Provincial Cancer Hospital (China). These specimens were frozen and stored in liquid nitrogen before analysis. Each patient was diagnosed as primary HCC without any treatment prior to surgical resection. This work gained the approval of the Ethics Committee of The Affiliated Bozhou Hospital of Anhui Medical University (2022).

### RNA extraction and reverse transcription quantitative real-time polymerase chain reaction

Total RNA was extracted by use of TRIzol reagent (St. Louis, Missouri, USA: St. Louis, Missouri, USA: Sigma-Aldrich). RNA content was tested utilizing a spectrophotometer. Samples with 260/280 absorbance ratio > 2 ± 0.1 were removed. Afterward, RNA was reverse transcribed into cDNA *via* reverse transcription kit (Dalian, China: Takara), followed by quantitative polymerase chain reaction (qPCR) by SYBR Premix Ex Taq (Dalian, China: Takara). The relative expression was computed with 2^-ΔΔCt^. The primers included LRPPRC, 5′-GCTCATAGGATATGGGACACACT-3′ (forward), 5′-CCAGGAAATCAGTTGGTGAGAAT-3′ (reverse); PD-L1, 5′-TGGCATTTGCTGAACGCATTT-3′ (forward), 5′-TGCAGCCAGGTCTAATTGTTTT-3′ (reverse); β-actin, 5′-CATGTACGTTGCTATCCAGGC-3′ (forward), 5′-CTCCTTAATGTCACGCACGAT-3′ (reverse).

### Western blot

Total protein was prepared utilizing RIPA reagent, followed by quantification with BCA assay kit (St. Louis, Missouri, USA: Sigma-Aldrich). Next, the sample was separated *via* SDS-PAGE gel kit (Wuhan, China: Elabscience). Being transferred onto PVDF membrane, the membrane was exposed to LRPPRC (1:2000; 21175-1-AP; Wuhan, China: Proteintech), β-actin (1:5000; 81115-1-RR; Wuhan, China: Proteintech), or PD-L1 (1:300; 28076-1-AP; Proteintech) primary antibody at 4°C overnight. Next, incubation with secondary antibody (1:5000; SA00001-2; Wuhan, China: Proteintech) was conducted at room temperature lasting 2h. The blots were imaged with enhanced chemiluminescence system, which were quantified *via* ImageJ software.

### Immunohistochemical staining

The section was cut into 3-μm thickness on paraffin-embedded tissue specimens. After 4h heat at 50°C, deparaffinizion and rehydration were implemented by the use of 100% xylene along with a gradient of ethanol. The activity of endogenous peroxidase was sealed utilizing 0.3% hydrogen peroxide lasting 15 min, followed by antigen retrieval. After blocking non-specific binding, the section was exposed to 100-μl blocking buffer lasting 25 min. Afterward, primary antibody of LRPPRC (1:50; 21175-1-AP; Proteintech), PD-L1 (1:500; 28076-1-AP; Proteintech), CD8 (1:200; 66868-1-Ig; Proteintech), CD4 (1:450; 67786-1-Ig; Proteintech), CXCL9 (1:50; 22355-1-AP; Proteintech), or CXCL10 (1:50; 10937-1-AP; Proteintech) was added to the section. After incubation overnight at 4°C, it was exposed to secondary antibody lasting half an hour at room temperature. Immunostaining was carried out by the use of the Envision System with diaminobenzidine.

### Cell culture

Human HCC-derived cell lines HepG2, Hep3B were acquired from the American Type Culture Collection (Manzas, Virginia, USA). These cells were maintained in Dulbecco’s modified Eagle’s medium (DMEM; Thermo Fisher Scientific) plus 10% fetal bovine serum (FBS; Sigma-Aldrich) and 1% penicillin/streptomycin (Sigma-Aldrich) in a 5% CO_2_ environment at 37°C.

### Transfection

The lentivirus interference vector LV-1 (pGLVU6/GFP) (GenePharma) was utilized for the expression of shRNAs against LRPPRC (sh-LRPPRC). Lentivirus was produced following the manufacturer’s protocols. The virus was utilized for infecting cells under 8 μg/ml protamine sulfate.

### EdU staining

Cells were planted into a 96-well plate (5 × 10^3^ cells per well). Cellular proliferation was measured by use of Cell-Light™ EdU Apollo488 Imaging kit (Guangzhou, China: RiboBio) in line with the manufacturer’s instructions.

### Flow cytometric analysis

Cells were inoculated into a six-well plate (3 × 10^5^ cells per well). Flow cytometric analysis was adopted for measuring the cell cycle distribution. In brief, cells were exposed to 5 μg/ml propidium iodide (PI) at 4°C away from the light. Following half an hour, the cellular DNA content was tested *via* flow cytometer (BD FACS Calibur). In addition, the cell percentage of distinct phases was assessed *via* FlowJo software.

### TUNEL staining

After dewaxing, the slides were rehydrated with ethanol, and exposed to 20 μg/ml protease K at 37° lasting 20 min, followed by administration with endogenous peroxidase blocking reagent at room temperature lasting 20 min. TUNEL (Terminal Deoxynucleotidyl Transferase mediated dUTP Nick-End Labeling) experiment was carried out on fixed cells by use of TUNEL (Terminal Deoxynucleotidyl Transferase mediated dUTP Nick-End Labeling) apoptosis detection kit (California, USA: Abbkine). Mount medium with 4′,6-diamidino-2-phenylindole (DAPI) was adopted for mounting the slides. The stained slides were photographed by use of a ZEISS microscope.

### Wound healing assay

Cells were seeded onto a six-well plate. When the cells were grown to 90% confluence, a 200-μl pipette tip was utilized for making a wound. Afterward, each plate was washed with 1× PBS buffer for discarding the detached cells and cultured with serum-free medium. Wound healing images at 0h and 48h were photographed, and migration rate was then calculated.

### Transwell assay

Matrigel (BD Biosciences) and serum-free medium were mixed at a 1:6 ratio and added to Transwell chamber (8 μm; Waltham, USA: Thermo Fisher Scientific). Following 1h, the suspended cells (1 × 10^5^) were seeded onto the upper chamber containing serum-free medium, and the medium with FBS (700 μl) was added to the lower chamber. At 24h, cells in the upper chamber were discarded. The invasive cells were fixed in 4% polyoxymethylene and dyed with crystal violet.

### RNA immunoprecipitation

This assay was conducted utilizing Magna RIP™ RNA-binding protein immunoprecipitation kit (Sigma-Aldrich). Cells were dissolved in 100% RNA immunoprecipitation (RIP) lysis buffer containing proteinase and RNase inhibitors, followed by exposure to RIP buffer comprising magnetic beads conjugated to anti–PD-L1 or IgG antibodies (Proteintech). Following 24h, RNA/bead complexes were resuspended in buffer composed of RNase-free DNase and proteinase K. RT-qPCR was implemented on the immunoprecipitated RNA for detect the enrichment.

### MeRIP−qPCR

Total RNA extracted by Trizol reagent were exposed to RNase-free DNase I (Waltham, USA: Thermo Fisher Scientific) for depleting DNA contamination, followed by purification and fragmentation of PolyA RNA. Afterward, 200 μg fragmented RNA was exposed to 3 μg anti-m^6^A (Synaptic Systems) within RIP reagent lasting 2h at 4°C and protein A/G magnetic beads for additional 2h. Moreover, 50 μl of immunoprecipitation reagent (Thermo Fisher Scientific) was utilized for eluting RNA. RT-qPCR was implemented on the immunoprecipitated RNA for detect the enrichment.

### Global m^6^A quantification

Global m^6^A level was measured utilizing EpiQuik™ m^6^A RNA Methylation Quantification Kit (Shanghai, China: Epigentek). After combining 200-ng RNA with captured antibody in each well, which was used for subsequent detection, m^6^A level was tested utilizing colorimetric approach at 450 nm and computed in line with the standard curves.

### RNA stability assay

Cells were seeded onto a six-well plate and exposed to 5 μg/ml actinomycin D (Shanghai, China: AbMole) for 0h, 2h, 4h, or 6h. Extracted RNA from the cells was utilized for RT-qPCR.

### Immunofluorescent staining

Cells were planted onto glass slides at 37°C lasting 24h and fixed with 4% paraformaldehyde for half an hour. The slides were incubated with anti–PD-L1 (1:50; 28076-1-AP) antibody for 20 min at room temperature away from the light. Mount medium with DAPI was utilized for mounting the slides. The stained slides were photographed utilizing a ZEISS microscope.

### Animal experiment

All animal care and procedures followed the National Institutes of Health Guidelines for Laboratory Animal Care. Male BALB/c nude mice (18–20 g weight, 6-week-old) were acquired from Beijing Vital River Laboratory Animal Technology Co., Ltd. (China). Xenograft tumors were produced through subcutaneously injecting 2×10^5^ control or sh-LRPPRC-transfected HepG2 cells under the arm. Tumor growth was measured by use of slide caliper every 3 days. Tumor volume was computed utilizing the formula 1/2 × (length × width^2^). Following three weeks, all mice were euthanized, the tumors were gathered for immunoblotting or immunohistochemical staining. This animal study gained the approval of the Animal Ethics Committee of The Affiliated Bozhou Hospital of Anhui Medical University (2022).

### Statistical analysis

All experimental data were analyzed by use of GraphPad Prism 8.0.1. Comparisons between groups were implemented *via* unpaired Student’s *t*-test, or one- or two-way ANOVA. Through Pearson’s correlation test, correlation analysis was carried out. *P* < 0.05 indicated statistical significance.

## Results

### LRPPRC is frequently upregulated in HCC and correlates to patients’ tumor staging and prognosis

**Figure 1:**
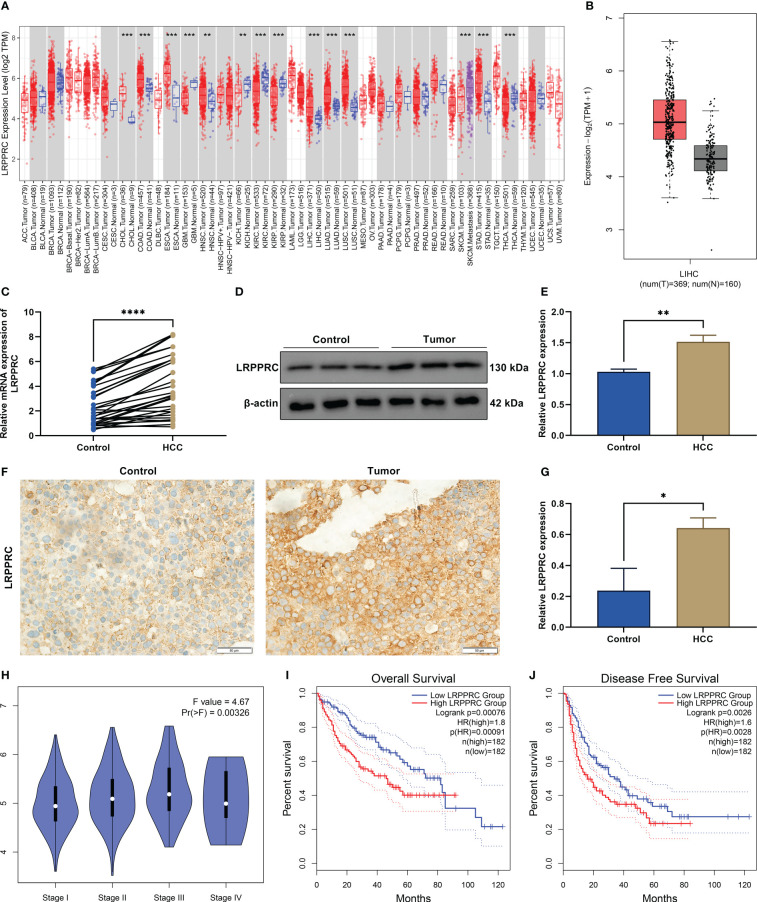
Leucine rich pentatricopeptide repeat containing (LRPPRC) presents the frequent upregulation in hepatocellular carcinoma (HCC) and correlates to patients’ tumor staging and prognosis. **(A)** The transcript level of LRPPRC in The Cancer Genome Atlas (TCGA) pan-cancer and matched normal tissues. **(B)** LRPPRC level in HCC and control specimens across TCGA-LIHC patients. **(C)** RT-qPCR of the transcript level of LRPPRC in paired HCC and control tissues. **(D)** Representative immunoblotting images of LRPPRC expression in such kinds of tissues. **(E)** Quantification of LRPPRC expression in line with the immunoblotting gray value. **(F)** Representative immunohistochemical staining photographs of LRPPRC in HCC and controls. **(G)** LRPPRC expression quantification in above tissues. **(H)** Difference in the transcript level of LRPPRC among distinct tumor stages of TCGA-LIHC patients. **(I**, **J)** Overall survival and disease-free survival probabilities of patients with lowly or highly expressed LRPPRC. *p < 0.05; **p < 0.01; ***p < 0.001; ****p < 0.0001.

In most cancer types, LRPPRC exhibited the notable upregulation in tumors relative to normal tissues (**Figure 1A**). Especially, it was frequently overexpressed in HCC tumors (**Figure 1B**). Further verification was conducted for such bioinformatics analysis findings. Thirty HCC and normal specimens were gathered. As expected, upregulated LRPPRC was confirmed in HCC *versus* controls in accordance with RT-qPCR (**Figure 1C**), immunoblotting (**Figures 1D, E** **)**, and immunohistochemical staining (**Figures 1F, G** **)**. In addition, LRPPRC presented the higher level in advanced stage across The Cancer Genome Atlas (TCGA)-Liver Hepatocellular Carcinoma (LIHC) patients (**Figure 1H**). This was indicative of the possible role of LRPPRC in HCC progression. The prognostic relevance was then assessed. With the median expression of LRPPRC, we categorized TCGA-LIHC patients as lowly or highly expressed LRPPRC subsets. As illustrated in **Figures 1I, J**, highly expressed LRPPRC subset possessed the worse overall survival as well as disease-free survival relative to another subset, indicating the contribution of LRPPRC overexpression to poor prognostic outcomes.

### LRPPRC suppression mitigates proliferative capacity and delays cell cycle progression in HCC cells

**Figure 2:**
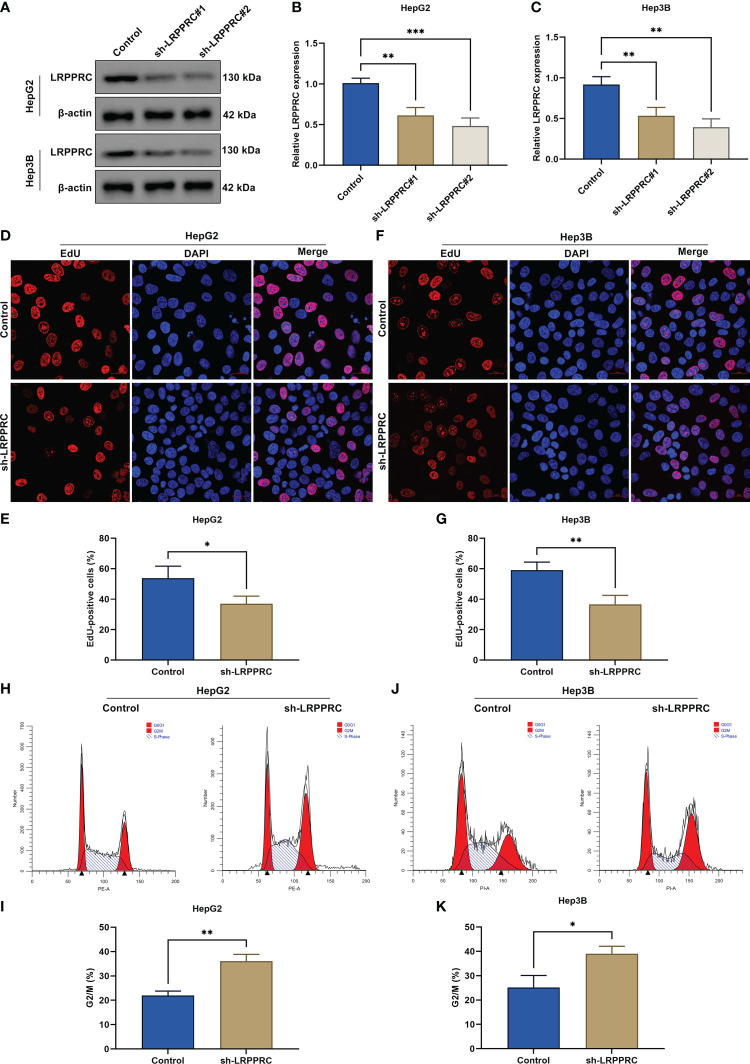
Leucine rich pentatricopeptide repeat containing (LRPPRC) suppression mitigates proliferative capacity as well as delays cell cycle progression in hepatocellular carcinoma (HCC) cells. **(A–C)** Immunoblotting of LRPPRC expression in HepG2 and Hep3B cells after LRPPRC was knockout. **(D–G)** EdU staining for the evaluation of proliferative ability of HCC cell lines with LRPPRC deficiency. Bar, 20 μm. **(H–K)** Flow cytometric analysis for the cell cycle distribution of LRPPRC-knockout HCC cells. *p < 0.05; **p < 0.01; ***p < 0.001.

For the assessment of LRPPRC function during HCC progression, its expression was effectively knockout in HepG2 and Hep3B cells through transfection of specific shRNAs (**Figures 2A–C**). Afterward, cellular proliferative capacity was measured *via* carrying out EdU staining. In comparison with controls, EdU-positive cells presented the notable reduction in LRPPRC-knockout cells (**Figures 2D-G**). This proved that LRPPRC was capable of affecting HCC proliferation. In addition, cell cycle distribution was tested by use of flow cytometric analysis. The proportion of G2/M was prominently elevated by LRPPRC deficiency in two HCC cell lines (**Figures 2H-K**), indicating the contribution of LRPPRC loss to G2/M cycle arrest of HCC.

### LRPPRC loss results in apoptosis of HCC cells and attenuates migratory and invasive abilities

**Figure 3:**
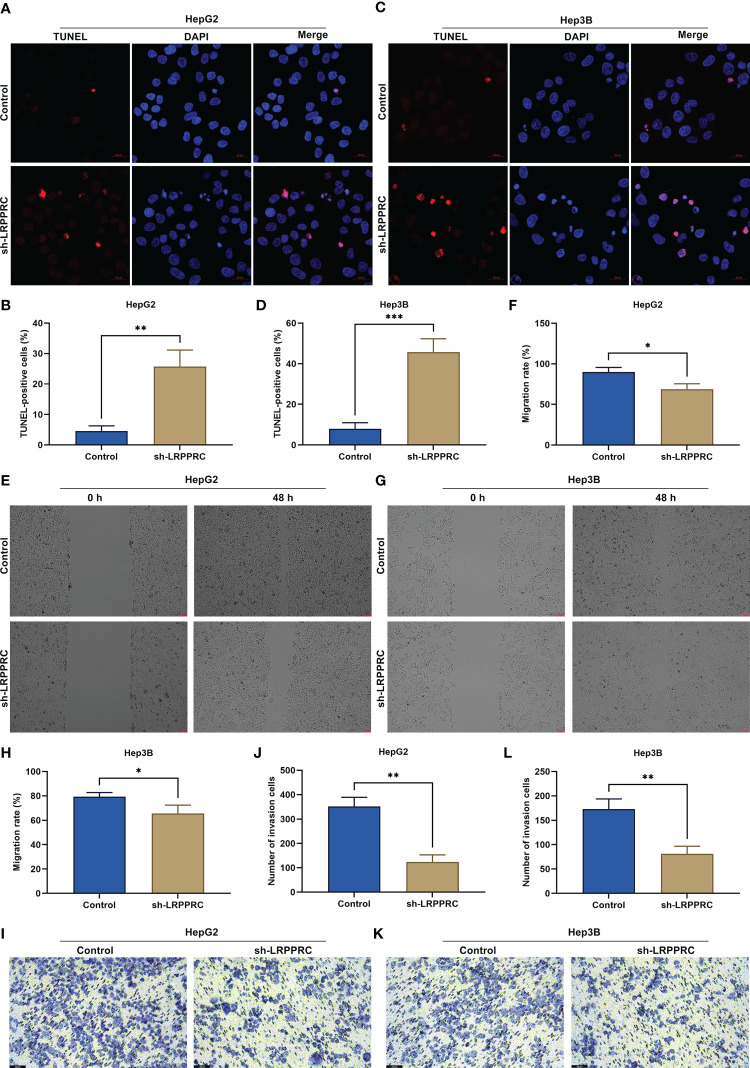
Leucine rich pentatricopeptide repeat containing (LRPPRC) loss results in apoptosis of hepatocellular carcinoma (HCC) cells and attenuates migratory and invasive abilities. **(A–D)** TUNEL (Terminal Deoxynucleotidyl Transferase mediated dUTP Nick-End Labeling) staining for the assessment of apoptotic level in LRPPRC-deficient HepG2 and Hep3B cells. Bar, 20 μm. **(E–H)** Wound healing test for investigating the migratory ability of HCC cells under the knockdown of LRPPRC. Bar, 50 μm. **(I–L)** Evaluation of the invasive capacity of LRPPRC-knockout HCC cells by use of transwell experiment. Bar, 100 μm. *p < 0.05; **p < 0.01; ***p < 0.001.

In accordance with TUNRL staining results, after LRPPRC was knockout, TUNEL (Terminal Deoxynucleotidyl Transferase mediated dUTP Nick-End Labeling)-positive HepG2 and Hep3B cells exhibited the prominent reduction (**Figures 3A-D**). This showed that targeting LRPPRC can induce HCC apoptosis. As shown in wound healing experiment, migratory level in HCC cells was remarkably mitigated by LRPPRC deficiency (**Figures 3E-H**). In addition, the impairment of invasive ability was observed in LRPPRC-knockout HCC cells (**Figures 3I-L**). Such findings were indicative that LRPPRC inhibition resulted in HCC cell apoptosis as well as impaired migratory and invasive capacities.

### LRPPRC presents a negative association with anti-tumor immunity and immune infiltration in HCC

**Figure 4:**
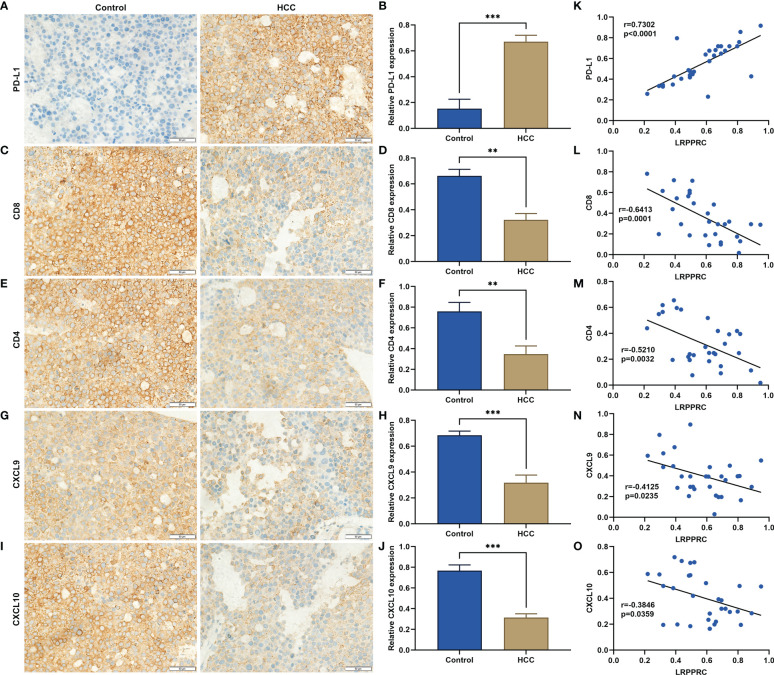
Leucine rich pentatricopeptide repeat containing (LRPPRC) exhibits a negative correlation to anti-tumor immunity and immune infiltration in hepatocellular carcinoma (HCC). **(A–J)** Representative immunohistochemical staining photographs and quantification results of **(A, B)** PD-L1, **(C, D)** CD8, **(E, F)** CD4, **(G, H)** CXCL9, **(I, J)** CXCL10 in 30 paired HCC, and normal tissues. **(K–O)** Scatter plots illustrating the relationships of LRPPRC with **(K)** PD-L1, **(L)** CD8, **(M)** CD4, **(N)** CXCL9, and **(O)** CXCL10. **p < 0.01; ***p < 0.001.

Immunohistochemical staining demonstrated the remarkable upregulation of PD-L1 in HCC tumor versus control specimens (**Figures 4A, B** **)**. HCC tumors exhibited the notably lower density of CD8+ and CD4+ T-cell infiltration relative to normal tissues (**Figures 4C-F**). CXCL9 and CXCL10 can be generated by antigen-presenting cells (dendritic cells and macrophages) and by tumor cells. Therefore, the two chemokines were measured by use of immunohistochemical staining. As a result, such chemokines were lowly expressed in HCC in comparison with normal specimens (**Figures 4G-J**). Further analysis was indicative that LRPPRC exhibited a positive relationship with PD-L1 across HCC patients, while displayed negative correlations to CD8, CD4, CXCL9, and CXCL10 (**Figures 4K-O**). Above data preliminarily proved that LRPPRC was negatively associated with anti-tumor immunity and immune infiltration in HCC.

### LRPPRC elevates m^6^A modification of PD-L1 mRNA in HCC cells

**Figure 5:**
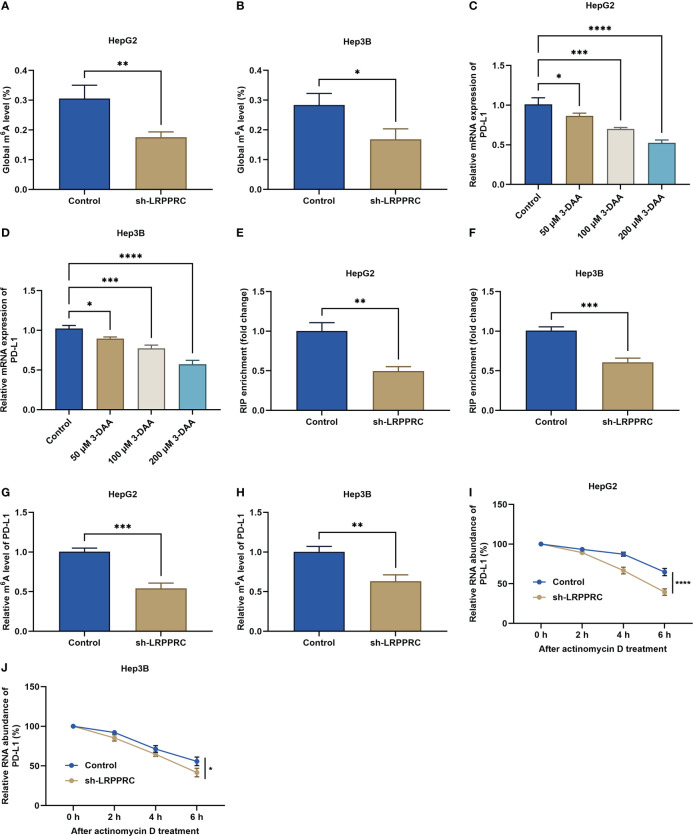
Leucine rich pentatricopeptide repeat containing (LRPPRC) elevates m^6^A modification of PD-L1 mRNA in hepatocellular carcinoma (HCC) cells. **(A**, **B)** the overall m^6^A modification in HepG2 and Hep3B cells with LRPPRC deficiency. **(C**, **D)** PD-L1 transcript level in LRPPRC-knockout HCC cells following 3-DAA exposure. **(E**, **F)** RIP-qPCR for the evaluation of the interactions of LRPPRC with PD-L1 mRNA in HCC cells with LRPPRC deficiency. **(G**, **H)** The relative m^6^A level in PD-L1 mRNA in LRPPRC-knockout HCC cells. **(I**, **J)** PD-L1 transcript level in LRPPRC-deficient HCC cells following actinomycin D administration. *p < 0.05; **p < 0.01; ***p < 0.001; ****p < 0.0001.

We firstly used the RM2Target platform (http://rm2target.canceromics.org/) to predict the m^6^A modification role of LRPPRC in PD-L1 (23). Our prediction data demonstrated that PD-L1 might be potentially modified by LRPPRC-mediated m^6^A modification. This work assessed the regulatory effect of LRPPRC on the overall m^6^A modification in HepG2 and Hep3B cells. Consequently, LRPPRC deficiency lowered the global m^6^A modification in two HCC cell lines (**Figures 5A, B** **)**. Moreover, it was found that PD-L1 mRNA level exhibited a prominent reduction by 3-deazaadenosine (3-DAA) methylation inhibitor with a concentration-dependent manner (**Figures 5C, D** **)**. RIP-qPCR was implemented for examining the effect of LRPPRC on posttranscriptional modification of PD-L1. It was found the prominently lower LRPPRC enrichment with PD-L1 mRNA *versus* IgG control (**Figures 5E, F** **)**. Based upon MeRIP-qPCR results, PD-L1 mRNA exhibited the remarkable reduction in m^6^A-modified level by LRPPRC deficiency (**Figures 5G, H** **)**. In addition, LRPPRC loss resulted in the decrease in mRNA stability of PD-L1, because the half-life of PD-L1 transcript was lowered under exposure to actinomycin D (**Figures 5I, J** **)**. Altogether, LRPPRC showed a direct interaction with PD-L1 and can modulate m^6^A modification of PD-L1 mRNA in HCC cells.

### LRPPRC upregulates PD-L1 expression in HCC cells partially with an m^6^A-independent manner

**Figure 6:**
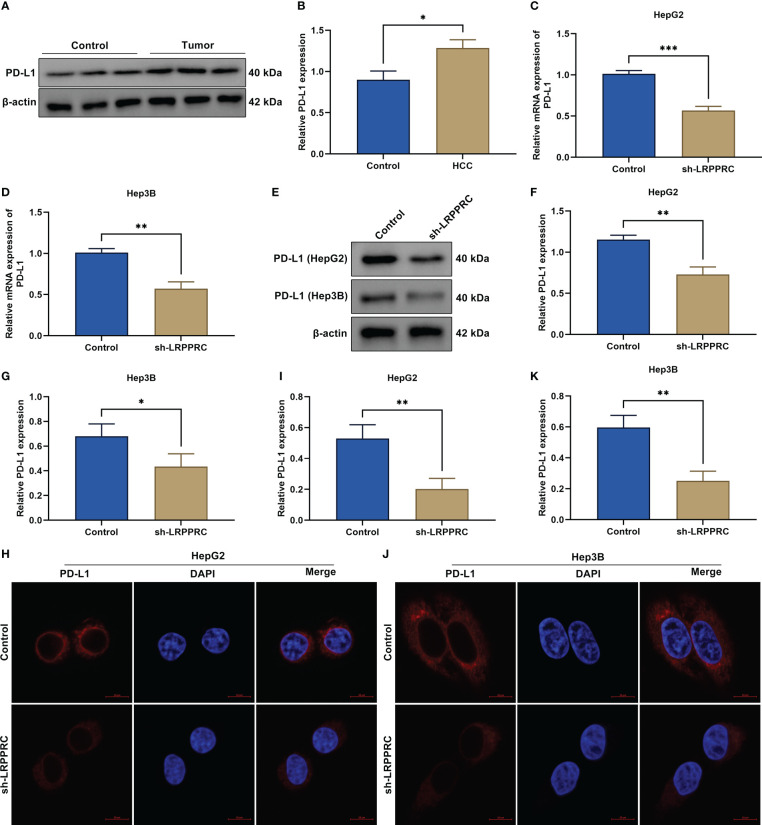
Leucine rich pentatricopeptide repeat containing (LRPPRC) upregulates PD-L1 expression in hepatocellular carcinoma (HCC) cells with an m^6^A-independent manner. **(A**, **B)** Immunoblotting of PD-L1 level in HCC tumors and control specimens. **(C**, **D)** Transcript level of PD-L1 in LRPPRC-knockout HepG2 and Hep3B cells. **(E–G)** PD-L1 protein level in HCC cells with LRPPRC loss. **(H–K)** Representative immunofluorescent staining photographs and quantification data of PD-L1 in LRPPRC-knockout HCC cell lines. Bar, 10 μm. *p < 0.05; **p < 0.01; ***p < 0.001.

As expected, PD-L1 exhibited the prominent upregulation in HCC tumors relative to control specimens (**Figures 6A, B** **)**. Its transcript and protein levels were notably decreased by LRPPRC deficiency in HepG2 and Hep3B cells (**Figures 6C-G**). Immunofluorescent staining also proved the reduction in PD-L1 protein level in LRPPRC-deficient HCC cells (**Figures 6H-K**). Such findings proved that LRPPRC enabled to upregulate PD-L1 expression in HCC cells partially with an m^6^A-independent manner.

### LRPPRC suppression mitigates tumor growth in HCC

**Figure 7:**
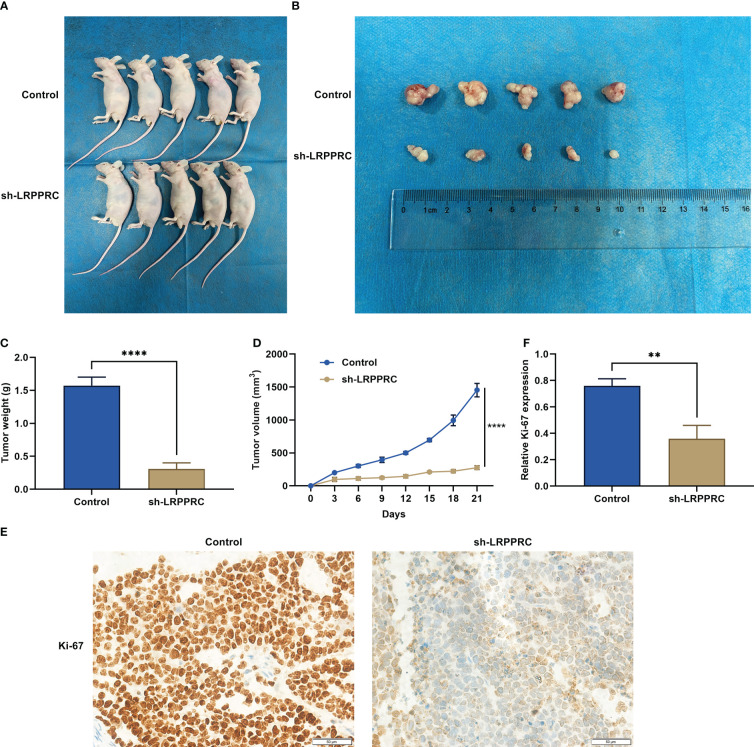
Leucine rich pentatricopeptide repeat containing (LRPPRC) suppression mitigates tumor growth in hepatocellular carcinoma (HCC). **(A)** Representative photographs of BALB/c nude mice injecting 2 × 10^5^ HepG2 cells with LRPPRC knockout or not. **(B)** Representative photographs of tumors from the indicated BALB/c nude mice. **(C**, **D)** Tumor weight and tumor growth curves. **(E**, **F)** Representative photographs and quantified data of immunohistochemical staining of Ki-67 in tumors. Bar, 50 μm. **p < 0.01; ****p < 0.0001.

HepG2 cells with LRPPRC knockout or not were subcutaneously inoculated into BALB/c nude mice. Following 3 weeks, tumor specimens were dissected. It was observed that LRPPRC-deficient group exhibited the lower tumor weight (**Figures 7A-C**). In addition, tumor growth was notably slowed down by LRPPRC knockdown (**Figure 7D**). In addition, Ki-67–positive tumor cells displayed the remarkable reduction in LRPPRC-deficient group (**Figures 7E, F** **)**. Thus, LRPPRC downregulation may mitigate tumor growth in HCC.

### LRPPRC loss improves anti-tumor immunity and immune infiltration *in vivo*

**Figure 8:**
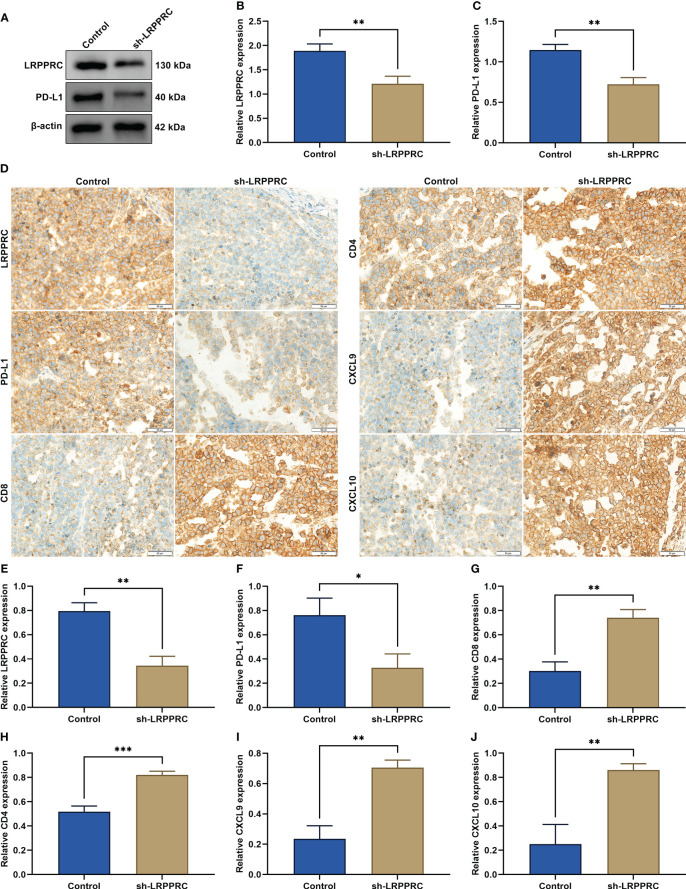
Leucine rich pentatricopeptide repeat containing (LRPPRC) loss improves anti-tumor immunity and immune infiltration *in vivo*. **(A–C)** Immunoblotting of LRPPRC and PD-L1 expression in tumors from BALB/c nude mice injected with LRPPRC-knockout or not 2 × 10^5^ HepG2 cells. **(D)** Representative immunohistochemical staining photographs of LRPPRC, PD-L1, CD8, CD4, CXCL9, and CXCL10 in tumor specimens. Bar, 50 μm. **(E–J)** Quantified data of **(E)** LRPPRC, **(F)** PD-L1, **(G)** CD8, **(H)** CD4, **(I)** CXCL9, and **(J)** CXCL10 in tumors in accordance with immunohistochemical staining. *p < 0.05; **p < 0.01; ***p < 0.001.

In the subcutaneous xenograft murine models, LRPPRC presented the remarkable downregulation in LRPPRC-knockout group (**Figures 8A, B** **)**. In addition, PD-L1 level was notably attenuated by LRPPRC deficiency in tumors (**Figure 8C**). Immunohistochemical staining also proven the downregulation of LRPPRC and PD-L1 in the murine models injected with LRPPRC-knockout HepG2 cells (**Figures 8D-F**). Moreover, CD8+ and CD4+ T cells exhibited the higher infiltration in tumors from LRPPRC-knockout group (**Figures 8G, H** **)**. The downregulation of CXCL9 and CXCL10 was also observed in this group (**Figures 8I, J** **)**. Altogether, LRPPRC deficiency strengthened anti-tumor immunity and immune infiltration *in vivo*.

## Discussion

HCC remains a dominating global healthcare challenge (24). Elements within the immune system exert an essential role in fighting tumor cells (25). Although such elements make the determined efforts into tumor elimination, tumor cells skillfully evade the immune system’s monitoring process *via* employing a variety of immune escape mechanisms, especially immunosuppression (26). Immune checkpoint inhibitors are emerging as a potent therapeutic option. Nonetheless, regulating the immune system with immune checkpoint inhibitors still faces serious immunogenic side effects and limited response (8, 27). Hence, the development of strategies to stimulate anti-tumor immunity may bring novel perspectives for HCC therapy.

Both in TCGA-LIHC and our cohorts, LRPPRC exhibited the frequent upregulation in HCC tumors, which was in relation to advanced stage as well as poor prognostic outcomes, consistent with previous findings (15, 16). Both *in vitro* and murine models, LRPPRC suppression was capable of attenuating malignant behaviors of HCC cells. Our evidence proposed LRPPRC as a possible therapeutic target against HCC. However, as an m^6^A modification reader, the role of LRPPRC in modulating m^6^A modification remains indistinct.

PD-L1 is a main co-inhibitory immune checkpoint and the PD1/PD-L1 signaling is capable of mitigating the killing role of cytotoxic T cells within the tumor microenvironment, thus contributing to tumor immune evasion (28, 29). Hence, further research on the regulatory mechanisms of PD-L1 in HCC is required. This work demonstrated that LRPPRC upregulated PD-L1 mRNA in HCC with an m^6^A-independent manner. In addition, both in HCC patients and murine models, LRPPRC exhibited a positive interaction with PD-L1, with negative correlations to CD8+, and CD4+ T-cell infiltrations and chemokines CXCL9, and CXCL10, indicating the possible role of LRPPRC in modulating anti-tumor immunity and immune infiltration. The m^6^A modification of PD-L1 have been reported. For instance, METTL3 posttranscriptionally upregulates PD-L1 expression in an m^6^A-IGF2BP3–mediated manner for enhancing stabilization of PD-L1 mRNA in breast carcinoma (30). In bladder carcinoma, JNK pathway facilitates immune evasion through upregulating METTL3-independnet m^6^A modification of PD-L1 (31). Tumor-intrinsic ALKBH5 attenuates the expansion and cytotoxicity of T cells through maintaining PD-L1 expression with YTHDF2-independnet m^6^A modification in intrahepatic cholangiocarcinoma (32). ALKBH5 is capable of facilitating the recruitment of PD-L1+ macrophages as well as accelerating HCC growth and metastases (33). In murine models, YTHDF1 deficiency can enhance antigen-specific CD8+ T-cell anti-tumor response as well as improve the therapeutic efficacy of anti–PD-L1 antibody (34). Our work proposed a novel mechanism of LRPPRC in mediating m^6^A modification of PD-L1 mRNA during HCC, which might further the present molecular understanding of immunosuppression and offer more effective immunotherapeutic regimens.

Altogether, our work on LRPPRC-mediated m^6^A modification of PD-L1 mRNA and anti-tumor immunity offered a new mechanism for m^6^A regulator-mediated immunosuppression in HCC. Thus, LRPPRC might possess a possible application as a new therapeutic target in combined with immunotherapy.

## Conclusion

Collectively, this work uncovered the m^6^A modification role of LRPPRC in PD-L1 mRNA stabilization in HCC cells and broadened the knowledge of a novel posttranscriptional regulation mechanism of PD-L1 expression and the functional significance of LRPPRC in anti-tumor immunity. This may have a possible implication for a novel and effective treatment option in HCC immunotherapy.

## Data availability statement

The original contributions presented in the study are included in the article/supplementary material. Further inquiries can be directed to the corresponding author.

## Ethics statement

The studies involving human participants were reviewed and approved by The Affiliated Bozhou Hospital of Anhui Medical University (2022). The patients/participants provided their written informed consent to participate in this study. The animal study was reviewed and approved by The Affiliated Bozhou Hospital of Anhui Medical University (2022).

## Author contributions

HL conceived and designed the study. HW, AT conducted most of the experiments and data analysis, and wrote the manuscript. YC, HG participated in collecting data and helped to draft the manuscript. All authors contributed to the article and approved the submitted version.
